# Phytogenic Additives Can Modulate Rumen Microbiome to Mediate Fermentation Kinetics and Methanogenesis Through Exploiting Diet–Microbe Interaction

**DOI:** 10.3389/fvets.2020.575801

**Published:** 2020-11-12

**Authors:** Faiz-ul Hassan, Muhammad Adeel Arshad, Hossam M. Ebeid, Muhammad Saif-ur Rehman, Muhammad Sajjad Khan, Shehryaar Shahid, Chengjian Yang

**Affiliations:** ^1^Key Laboratory of Buffalo Genetics, Breeding and Reproduction Technology, Ministry of Agriculture and Guangxi Buffalo Research Institute, Chinese Academy of Agricultural Sciences, Nanning, China; ^2^Institute of Animal and Dairy Sciences, Faculty of Animal Husbandry, University of Agriculture, Faisalabad, Pakistan; ^3^Dairy Science Department, National Research Centre, Giza, Egypt

**Keywords:** rumen, microbiome, methane, fermentation, VFA, plant secondary metabolites

## Abstract

Ruminants inhabit the consortia of gut microbes that play a critical functional role in their maintenance and nourishment by enabling them to use cellulosic and non-cellulosic feed material. These gut microbes perform major physiological activities, including digestion and metabolism of dietary components, to derive energy to meet major protein (65–85%) and energy (ca 80%) requirements of the host. Owing to their contribution to digestive physiology, rumen microbes are considered one of the crucial factors affecting feed conversion efficiency in ruminants. Any change in the rumen microbiome has an imperative effect on animal physiology. Ruminal microbes are fundamentally anaerobic and produce various compounds during rumen fermentation, which are directly used by the host or other microbes. Methane (CH_4_) is produced by methanogens through utilizing metabolic hydrogen during rumen fermentation. Maximizing the flow of metabolic hydrogen in the rumen away from CH_4_ and toward volatile fatty acids (VFA) would increase the efficiency of ruminant production and decrease its environmental impact. Understanding of microbial diversity and rumen dynamics is not only crucial for the optimization of host efficiency but also required to mediate emission of greenhouse gases (GHGs) from ruminants. There are various strategies to modulate the rumen microbiome, mainly including dietary interventions and the use of different feed additives. Phytogenic feed additives, mainly plant secondary compounds, have been shown to modulate rumen microflora and change rumen fermentation dynamics leading to enhanced animal performance. Many *in vitro* and *in vivo* studies aimed to evaluate the use of plant secondary metabolites in ruminants have been conducted using different plants or their extract or essential oils. This review specifically aims to provide insights into dietary interactions of rumen microbes and their subsequent consequences on rumen fermentation. Moreover, a comprehensive overview of the modulation of rumen microbiome by using phytogenic compounds (essential oils, saponins, and tannins) for manipulating rumen dynamics to mediate CH_4_ emanation from livestock is presented. We have also discussed the pros and cons of each strategy along with future prospective of dietary modulation of rumen microbiome to improve the performance of ruminants while decreasing GHG emissions.

## Introduction

Improving feed efficiency and livestock production is a more coveted goal in animal agriculture being sought through selective breeding, scientific management, and improvement of feed composition. Feed efficiency in ruminants mainly depends upon the quality of feed, rumen fermentation, and dynamics mediated by rumen microbiomes. The rumen in animals is inhabited by the diverse microbiome, including bacteria, protozoa, fungi, and archaea. Different factors like temperature (38–42°C), pH (5.5–7), and redox potential (250–450 mV) regulated by saliva buffering provide a specific environment for degradation of cellulolytic plant material by microbes (1).

Degradation of various feed components is being accomplished by mutual interaction of microbiota to yield mainly acetate, propionate, butyrate, hydrogen (H_2_), carbon dioxide (CO_2_), and ammonia (NH_3_). Total VFA (75% of total amount) are the primary source of energy for the animal (2). Besides, microbial cell biomass is also utilized as the primary origin of protein and amino acids by host animals (3). The microbial ecosystem likewise produces vitamins B and K and utilizes the products of phytotoxin and mycotoxin detoxification processes (4). The ingested fiber is mainly degraded by bacteria and fungi into soluble nutrients (5). These soluble nutrients are subsequently used for the maintenance, growth, production, and reproduction of animals. During rumen fermentation of feed, some by-products are additionally produced, such as CO_2_ and H_2_, which are further converted into CH_4_ by some methanogens like *Methanopyrales, Methanomicrobiales, Methanobacteriales, Methanococcales, Methanocellales*, and *Methanosarcinales*). Some archaea (*Methanoplasmatales* or *Thermoplasmatales*) can also form CH_4_ through other substrates, such as methanol and mono-, di-, and tri-methylamine (6–8). Major greenhouse gases (CH_4_ and CO_2_) are released during enteric fermentation from ruminants. Production of CH_4_ also deprives the host animal of carbon resources and results in loss of energy (13.3 Mcal/kg CH_4_), leading to poor feed efficiency (9). Maximizing the flow of metabolic hydrogen ([H]) in the rumen away from CH_4_ and toward VFA would increase the efficiency of ruminant production and decrease its environmental impact. Czerkawski (10) proposed that inhibiting methanogenesis could favor microbial biomass production as an alternative [H] sink. Chalupa (11) suggested that metabolic hydrogen incorporated into excess NADH was redirected to fatty acid synthesis and fermentation end products such as lactate and ethanol, although the latter sinks were not quantitatively important (12).

Owing to its diverse physiological and metabolic functions, the rumen microbiome is considered the ultimate target to improve the energetic efficiency of animals while reducing environmental hazards like CH_4_ emissions. Highly efficient animals produce less CH_4_ and produce more milk, consuming less feed owing to their unique set of rumen microbiome (13). Specific physiological processes in lactating animals are correlated with specific rumen microbes owing to their unique fermentation and metabolic activities (14). The association of VFA composition with rumen bacteria has been reported in dairy cows possessing different efficiencies of production (15). Moreover, the rumen microbiome varies significantly among different animals, but intra-animal variation in microflora is quite less (16). These facts indicate the crucial role of the rumen microbiome in shaping the physiology of digestion and production in lactating animals and its potential utility for manipulation of performance and health.

Greenhouse gases (GHG) produced from ruminants have been an area of environmental concern (17, 18). Improving animal production systems must understand societal concerns and should realize the effect of such systems on the environment (19). Increasing feed efficiency to enhance animal production should also focus on CH_4_ mitigation strategies to reduce GHG emissions. In this regard, identification and manipulation of the microbes associated with methanogenesis are considered a significant and most crucial step (19, 20). Methanogenesis occurs both in the rumen and hindgut, but 90% of the total CH_4_ production originates from the rumen (21). A better understanding of digestive physiology and feed fermentation in rumen is necessary to ensure further improvement of production efficiency in ruminants to overcome the increasing demand for food by growing the human population. To achieve this daring task, manipulation of rumen fermentation is required to increase feed conversion efficiency while decreasing energy losses in the form of CH_4_ emanations through dietary interventions (22).

Manipulation of rumen fermentation is considered as an optimization process to seek suitable conditions for maximization and/or minimization of the specific rumen fermentation pathways, depending on factors such as type and level of feeding and animal production. The basic target behind such manipulation is the alteration in ruminal microflora that can be achieved by dietary intervention and the use of additives that selectively affect rumen communities. Improvement in ruminant production is possible with the manipulation of rumen fermentation to increase total VFA and propionate production while decreasing CH_4_ emission through reducing rumen methanogenesis (23). Many feed additives such as antibiotics, ionophores, and defaunating agents have been utilized to mediate rumen fermentation to improve the productivity of ruminants and reduce methanogenesis. However, most chemical additives either are noxious to host animals or present a temporary impact on methanogenesis (24, 25). Therefore, nutritionists and microbiologists are continuously trying to explore some natural substances with anti-methanogenic activity for eco-friendly animal production by reducing CH_4_ emission and its greenhouse effects (26).

We need to strengthen our understanding of diet–microbe interactions to devise dietary interventions to modulate the rumen microbiome to improve production efficiency and reduce energy losses in the form of GHG emissions. Moreover, we need to explore natural feed additives with limited or no adverse effects to manipulate rumen fermentation to improve feed digestibility and utilization. Plant secondary metabolites are natural substances with the potential ability to alter rumen fermentation without causing microbial resistance, and their residual effects can positively affect the animal end products (27, 28). Owing to the excellent antimicrobial activities of phytochemicals, they are considered as a potential modulator of the rumen microbiome to alter rumen physiology (29). Many experiments, including both *in vitro* and *in vivo* studies, have been conducted to explore the potential of phytochemicals on rumen fermentation to increase feed digestibility and reduce methanogenesis (30–32). Many of them have shown promising results, but applicability in terms of efficient animal production is questionable. Therefore, efforts are still underway to find an appropriate feed additive to mitigate rumen CH_4_ production, simultaneously improving livestock production while reducing greenhouse effects on the environment. To accomplish this challenging task, an in-depth understanding of rumen development, microbial colonization, the interaction of rumen microbiome with the host, and diet is indispensable. Therefore, this review aims to provide insights into the effect of different phytogenic and dietary interventions on ruminal microbes to mediate rumen fermentation and methanogenesis to increase overall feed efficiency to make livestock production sustainable and more profitable.

## Ontogenesis of the Rumen and Initial Microbial Colonization

The ruminant digestive system switches from monogastric to become fully active post-weaning rumen with the ability to digest fibrous feed. During the suckling period of the calf, milk bypasses the rumen due to the esophageal groove. Developed rumen comprises 60–80% of the total digestive system as compared to the monogastric stomach in early life. Besides this, rumen villi are not yet developed, which are necessary for the absorption of nutrients (33, 34). Rumen microbial populations exhibit an incredible impact on rumen structure and physiological development. Initial inoculation of rumen microbes in calves constitutes both aerobic and facultative anaerobic microbial taxa following birth, which later on mostly are replaced by anaerobic taxa (35). That is why 1-day-old calves have a massively different bacterial population as compared to 3-days-old calves (14).

The oxidative condition of the rumen is a primary regulator of shifts in the newborn rumen ecosystem, and redox has an inert impact on the colonization of methanogenic species (36). Ruminal bacteria such as cellulolytic species, *Ruminococcus flavefaciens*, and *Ruminococcus albus* and members of the *Prevotella* genus can already be detected on day one after birth. These microbes are involved in various rumen functions, such as cellulose and hemicellulose degradation (14). One of the primary changes observed throughout the rumen development includes modification in configuration within the *Bacteroidetes* phylum. In the developed rumen, this phylum is dominated by the genus *Prevotella* across several ruminant species (37). Nevertheless, during the primary stages of development, *Bacteroides* is the main genus within *Bacteroidetes* and is subsequently replaced by the *Prevotella* during the first 2 months (38).

Quick fluctuations in community configuration also affect methanogenic archaeal communities along with bacteria. Rumen methanogenic communities in calves and lambs have been detected as early as 20 min after birth. Like bacterial populations, the primary methanogenic population varies significantly between young and adult animals (39–41). Both preweaning calves and mature animals have a *Methanobacteriales* order, but rumen of preweaning calves contains two additional orders, *Methanosarcinales* and *Methanomicrobiales* (41). The compositional variations in rumen archaea lead to shifts in substrate utilization, methanogenic pathway, and extent of CH_4_ production (42).

The establishment and colonization of microbiota play a key role in the development and function of the gastrointestinal tract (GIT), which is subsequently associated with higher body weight and feed efficiency of growing ruminants (43). Developing a rumen ecosystem during weaning age is key to getting improved growth rates and better health at a later stage of life (44, 45). The main objective of such strategies is to overcome the risk of undesirable health consequences associated with an altered gut microbiome in neonatal animals and restoration of the gut microbial community following dysbiosis. A complete understanding of early gut colonization is necessary to designing different effective strategies to manipulate the GIT microbiome. Although a wealth of literature is available on different aspects of rumen microbiome in adult animals and early colonization of gut microbiota, information regarding the role of host genetics and microbial interactions in the early development of the gut microbiota is limited.

## Modulation of Rumen Microbiome Using Phytogenic Feed Additives

Ruminants can transform fibrous and non-fibrous plant material into valuable products like meat and milk with the help of rumen microbes (46). Rumen inhabits various microbes like bacteria, protozoa, fungi, archaea, and bacteriophages (47). A symbiotic relationship exists between rumen microbes and the host animal in which both provide coveted substance to each other mainly in three ways: (1) mastication and rumination expand the surface area of feed particles for microbial attachment and digestion, and consequently, microbes secrete fibrolytic enzymes for degradation of cellulose, and hemicelluloses; (2) ruminal movements (peristalsis and antiperistalsis) bring microbes in contact with the fresh substrate by mixing of digesta and consequently yield fermentation products, especially VFA; and (3) elimination of fermentation products by belching and absorption is essential for keeping ideal conditions (pH) for microbial development and utilizing non-protein nitrogen (48).

Ruminal bacteria are the most prevailing microbiome, and their population measured by direct counts is usually 10^11^ cells per gram of rumen contents (4) comprising more than 200 species (49). Bacteria colonize inside rumen and have a major role in the metabolism of dietary carbohydrates and nitrogen and utilize fiber, starch, protein, and sugars. Generally, ruminal bacteria used homoserine lactone-based quorum sensing to communicate with each other (50). The most important genera of ruminal bacteria are *Butyrivibrio, Prevotella, Ruminococcus*, and *Pseudobutyrivibrio*. Mainly CH_4_ emissions depend upon the abundance of H_2_-producing bacteria in the rumen (51).

Ruminal fungi comprise 5–20% of the total microbiota in the rumen (52, 53). Anaerobic fungi are known as key players for the breakdown of lignocellulosic fiber (54). Anaerobic fungi are considered one of the most potent fiber-degrading agents, because of their active and extensive set of enzymes for the breakdown of plant polymers (55). Fungi produce enzymes vital for the digestion of plant materials, including cellulases, xylanases, mannanases, esterases, glucosidases, and glucanases (56). Rumen fungi also possess amylolytic (57) and proteolytic activities (58). The action of anaerobic fungi is promoted by the methanogenic archaea (59). However, the present understanding of rumen eukaryote function is far less than that of rumen bacteria, primarily due to the restricted annotation of the transcriptome and multiple-genome sequence availability (60). A recent *in vitro* study reported that a combination of anaerobic fungi (*Caecomyces*) and methanogens (*Methanobrevibacter*) have a greater ability to degrade lignocellulose and to produce CH_4_ as compared to the combination of bacteria and methanogens, and whole rumen content enrichment (61).

Ruminal protozoa represent about 20–50% of total microbial biomass and are commonly grouped into flagellates and ciliates. The flagellate proportion to overall ruminal fermentation is negligible (62). However, ciliate protozoa have a fundamental function in rumen fermentation as they engulf fermentable carbohydrates (63) and prevent alternative bacterial fermentation that would otherwise decrease pH and increase the onset of lactic acid acidosis (64). There is a positive correlation between ruminal protozoa and volatile fatty acid and CH_4_ production. Ciliate protozoa can enhance the metabolic output of the rumen microbiome; for instance, acetate, butyrate, iso-butyrate, and iso-valerate concentrations were improved in microcosms incubated with the protozoa population (65). The hydrogenosomes of rumen protozoa are involved in the production of H_2_, which is subsequently converted to CH_4_ by the methanogens through the hydrogenotrophic pathway (66, 67). Approximately 11% reduction in the CH_4_ output has been observed due to the defaunation of protozoa (64, 68).

**Figure 1:**
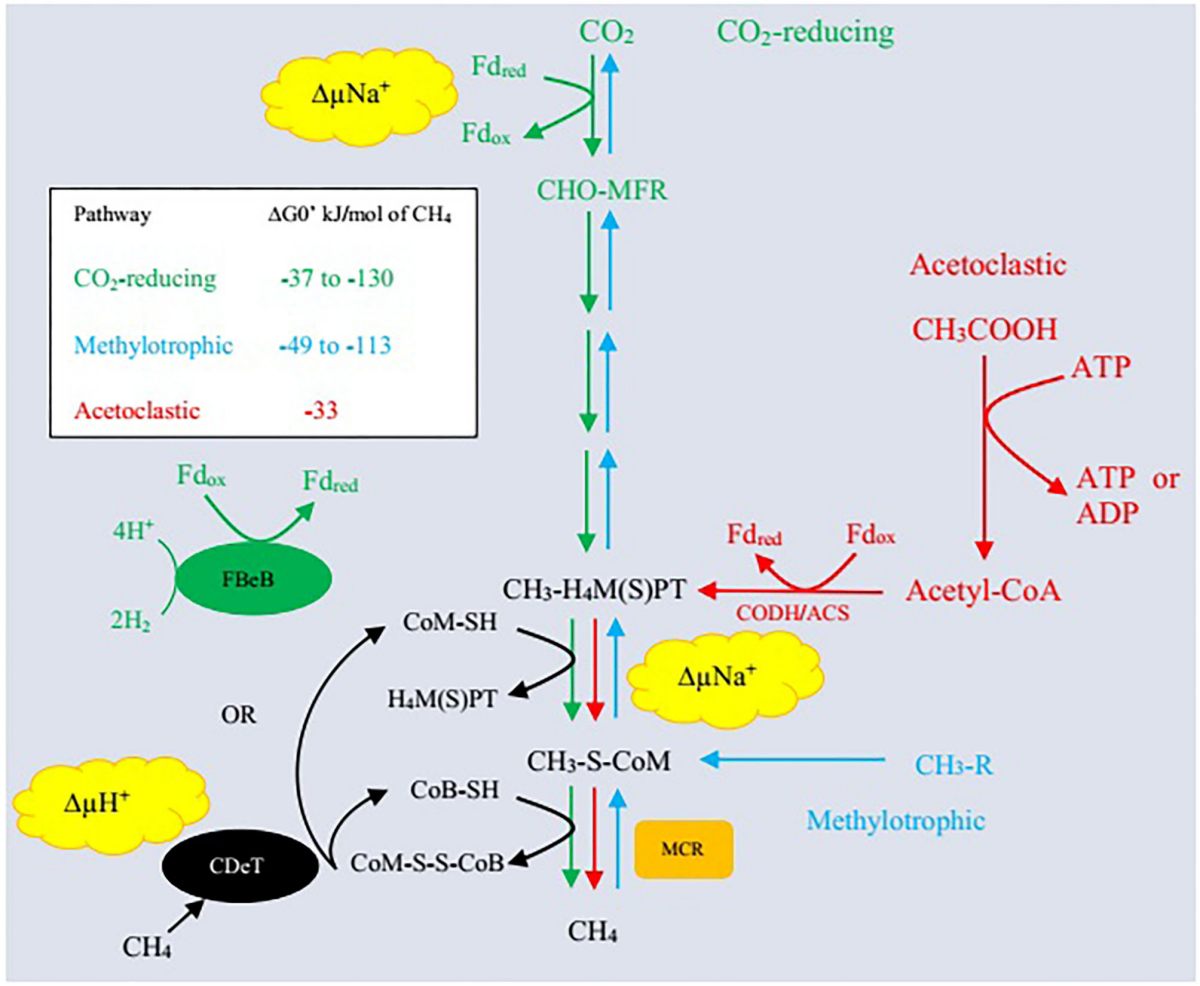
Three enzymatic pathways of methanogenesis. Δ, CO_2_-reducing pathway (hydrogenotrophic pathway); Δ, acetoclastic pathway; Δ, methylotrophic pathway; MFR, methanofuran; H_4_MPT, tetrahydromethanopterin; H_4_SPT, tetrahydrosarcinapterin; ΔG^0′^, standard free energy change; CH_3_-R, methyl-containing compounds such as methanol, methanethiol, dimethylsulfide, monomethylamine, dimethylamine, trimethylamine, and tetramethylammonium; Fd_red_, reduced form of ferredoxin; Fd_ox_, oxidized form of ferredoxin; ΔμNa^+^, electrochemical sodium ion potential; ΔμH^+^, electrochemical proton potential; FBeB, flavin-based electron bifurcation; CDeT, cytochrome-dependent electron transfer; MCR, methyl-coenzyme M reductase; CODH/ACS, carbon monoxide dehydrogenase/acetylCoA synthase/decarbonlyase complex. Adapted from Lyu et al. (72).

Archaea represent the third major domain of rumen microbes that constitute about 21% of the rumen microbiome (69). Methanogenic archaea belong to the phylum *Euryarchaeota* and are ubiquitously involved in methanogenesis (7, 69). Rumen methanogens have a synergistic association with bacteria and a symbiotic association with protozoa as <1% of the total microbial population (70). Different substrates are utilized during methanogenesis including formate, or acetate, methanol, H_2_, methylamines, and CO_2_ (71). Methane is produced mainly through three pathways: (i) primarily by reduction of CO_2_ through the hydrogenotrophic pathway, (ii) less from the use of methyl groups (methylotrophic pathway), and (iii) even less through acetate (acetoclastic pathway) production (Figure 1). Methanogenic paths comprise three stages: exchange of the methyl set to coenzyme M (CoM-SH), reduction of methyl-coenzyme M with coenzyme B (CoB-SH), and reuse of heterodisulfide CoM-S-S-CoB (51, 68, 73).

The bacteriophage community is also an important component of the rumen microbial ecosystem. Studies have reported inconsistent findings of bacteriophage counts ranging from >10^9^ particles of phages (74) to between 3 × 10^9^ and 1.6 × 10^10^ particles per ml of rumen content (75). Bacteriophages possess a specific lysogenic ability against different bacteria that helps in bacterial mass turnover in the rumen. Due to a lack of information regarding the mechanisms of rumen phage–host interactions and the environmental factors affecting the relative proportions and dynamics of the phage population in the rumen, it is not possible to definitively determine whether the presence of phage in the rumen is disadvantageous or advantageous. However, possible functional consequences of rumen phages have been proposed as (1) the negative nutritional consequences of phage-induced bacterial lysis resulting in the recycle of nutrients within the rumen, (2) the positive effects of maintaining bacterial population diversity and facilitating gene transfer, and (3) the negative consequences of phage-mediated gene transfer.

Keeping in view their critical role in digestive physiology and nutrient metabolism, modulation of the rumen microbiome is envisioned as a practical strategy to mediate fermentation kinetics and methanogenesis. Modulation of the rumen microbiome can be possible through different dietary interventions; however, in this regard plant secondary metabolites possess a greater potential as compared with antibiotics to modulate the ruminal microbiome and mitigate CH_4_ emission through diverse antimicrobial mechanisms such as perturbation of cell membrane, modulation of signal transduction or gene expression pathways, enzyme inhibition, and inhibition of bacterial colonization (76, 77). Plant secondary metabolites usually enhance the permeability and fluidity of the cellular membranes, further causing an efflux of metabolites and ions and ultimately leading to cell leakage and microbial death. Besides, they can also desirably manipulate the rumen metabolism by increasing the cell membrane's permeability of few specific rumen bacteria (78, 79). Putative mechanisms of actions mainly include disturbance of the cytoplasmic membrane, disruption of the proton motive force, electron flow, active transport mechanisms, and coagulation of cell composition (80).

## Genetic Manipulation of Rumen Microbiota

The host diet has a major influence on the relative abundance and diversity of the rumen microbiome. However, genetic manipulation of rumen microbiota is also possible through different techniques as host genetics influences some heritable microbial traits (81–83). In this regard, recent biological techniques such as transgenesis are getting attention for improving the efficiency of animal production and reducing environmental impacts (84). New genome editing tools provide an efficient way to produce gene-edited ruminants, having resistance against certain diseases and specific product quality (85). In transgenic animals usually, a foreign gene of interest is inserted into its genome to express a desirable trait (86). In a study of pig transgenesis, neomycin phosphotransferase transgene has been evaluated using high-throughput sequencing. Neo-transgenic expression in transgenic pigs showed a significant increase in the relative abundance of some bacteria (*Firmicutes, Bacteroidetes*, and *Proteobacteria*) with a reduction of potentially harmful bacteria such as *Escherichia–Shigella–Hafnia* (87). A recent study by Yang et al. (88) showed inactivation of the ABO acetyl-galactosaminyl-transferase gene through a deletion of 2.3 Kb, potentially affecting the microbiota composition and its relative abundance (particularly *Christensenellaceae* and *Erysipelotrichaceae* families).

Presently, different studies are being focused to identify heritable microbes and microbial features in humans and animals, considering gut microbiomes as heritable phenotypes, (89). Elucidation of the association between host genetics and rumen microbiome composition will help to identify persistent microbial taxa and functions that are characteristic of efficient animals, thereby directly facilitating efforts to genetically select or permanently alter the rumen microbiome. Within the next 5–10 years, significant progress is expected regarding the relationship between gut microbiomes and the genetics of their ruminant hosts, unraveling an intricate network that paves the way for the genetic selection of heritable microbes and keystone microbial species. These efforts will rapidly advance microbial ecology research and animal production to efficiently and sustainably produce high-quality protein for human consumption to considerably contribute to global food security (90).

## Diet–Microbe Interactions

**Figure 2:**
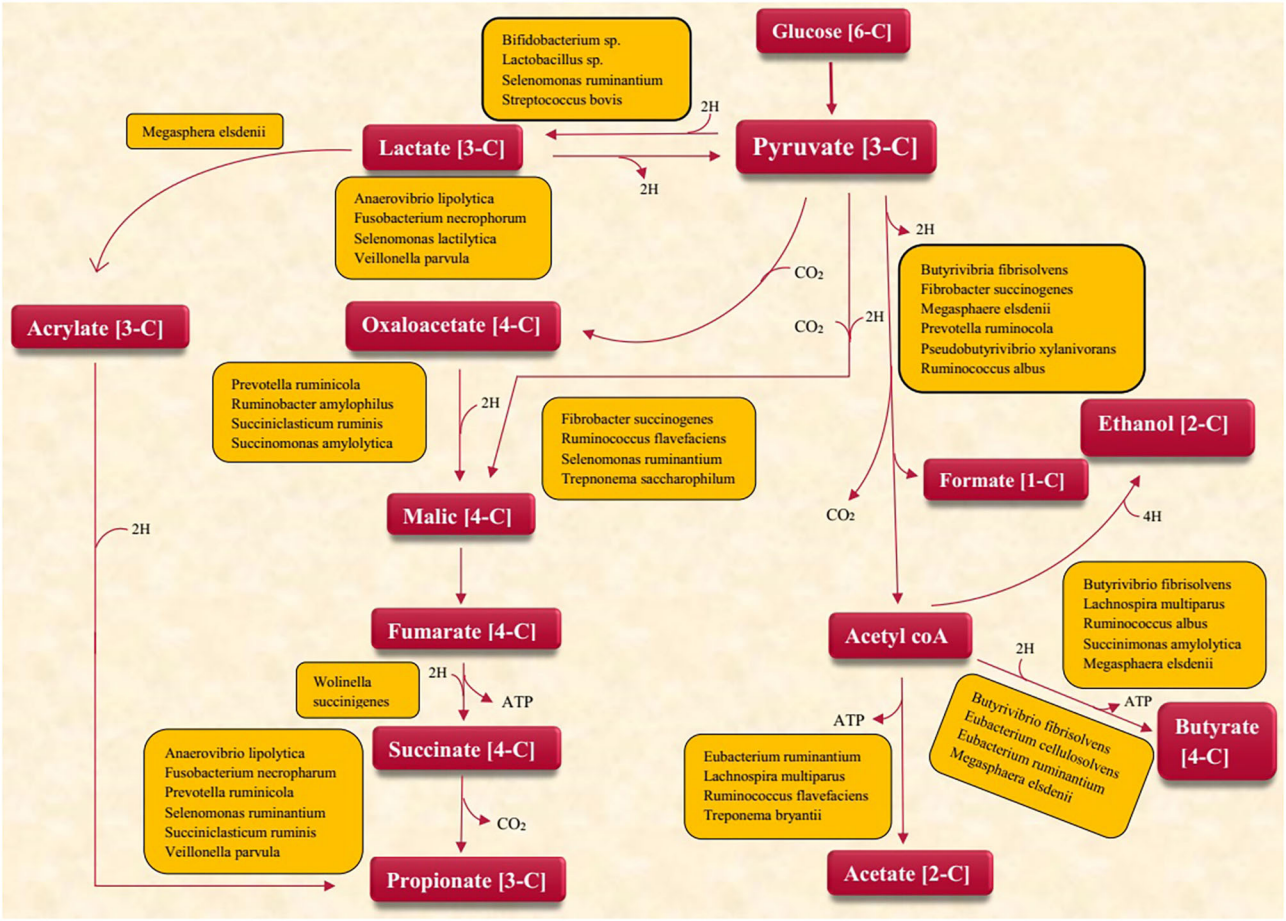
Microbes involved in VFA production.

Emerging feeding techniques to limit CH_4_ emissions are necessarily required both for preserving the environment and for increasing the efficacy of energy utilization. Different microbes are involved in the production of VFA (Figure 2). The rumen of dairy cow possesses diverse consortia of microbes that produce significant amounts of GHG gases (mainly CH_4_) during feed digestion (48). Methodologies to divert rumen carbon and nitrogen metabolism away from these products offer opportunities for improving the efficiency of ruminant production by enhancing nutrient utilization while reducing GHG emissions.

Many studies have been conducted regarding the development of nutritional interventions to cut down CH_4_ emissions from ruminants (91–93). With increasing food safety concerns, different natural compounds of plants are being considered ideal for moderating/mitigating CH_4_ emissions (94). The diet fed to ruminants is the primary determinant of bacterial community structure (95–97). Co-oscillations of microbiota are very important to maintaining homeostasis in gastrointestinal ecology after dietary perturbations (98). It is important to adjust the animal diet according to their age and physiological condition and to provide proper time to adapt to different dietary changes. Diet is also considered as an important factor to ensure proper animal health and performance because digestion and utilization of nutrients mainly depend upon collaboration and competition of the microbiome. Important diet–microbe interactions are discussed as below.

### Carbohydrate–Microbe Interactions

Rumen microbiology involves the characterization of microbes and processes associated with fiber digestion (17, 99, 100). Rumen bacteria play a dominant role in fiber digestion, although anaerobic fungi and protozoa have been reported to contribute to lignocellulose breakdown by attacking lignocellulosic material differently (60, 64) and discharging different enzyme complexes (101). Microbial communities found in the rumen of cows fed total mix ration (TMR) vary as compared to pasture-fed cows, owing to the variable dietary composition (102, 103). Different rumen bacteria have shown associations with specific diets like *Fibrobacteraceae* with TMR and *Veillonellaceae* with pasture-based diets (104).

Feeding concentrate diets have shown to lower the ruminal pH, increase VFA concentrations and osmolality, and induce metabolic disorders (105). Moreover, high starch feeding substantially increases the activity of lactic acid consuming and producing bacteria in the rumen because these microbes are not susceptible to lower pH and hence opportunistically utilize higher substrate availability (106). In contrast, feeding a considerable amount of roughages may limit feed consumption, energy efficiency, and microbial protein synthesis in ruminants (107). Therefore, an increasing quantity of starch in the diet is considered as a promising strategy to decrease methanogenesis per unit of dry matter intake through shifting ruminal fermentation toward propionogenesis (108). It is mainly attributed to the fact that the supply of substrates and microbial growth depends upon the dietary fiber or starch contents (109). Increased dietary starch levels have shown a relatively lower proportion of cellulolytic bacteria *R*. *albus* and *R. flavefaciens*, owing to their higher sensitivity toward low pH. However, in an acidic ruminal environment *F*. *succinogenes* have shown to remain stable due to their gram-negative nature and different cell membranes than *R. albus* and *R. flavefaciens* (110). Feeding grain-based diets to cattle could reduce the bacterial diversity compared to forage-based diet (111). This reduction might be attributed to less availability of substrates for bacteria that ferment structural carbohydrates and the subsequent lower pH.

Feeding of high starch and low fiber diets has also been shown to enhance the growth of amylolytic bacteria in the rumen. Propionate is produced by *S. ruminantium* through decarboxylation of succinate (6). This bacterium is capable of using starch and sugar for its growth. Higher dietary starch contents have been shown to increase the concentrations of propionic acid in the rumen substantially. Furthermore *S*. *ruminantium* is also capable of using lactic acid to stabilize the rumen pH (112). Thus, an increase in the population of these bacteria can enhance the utilization of fermentable substrates generated in the rumen following a high concentrate diet. A facultative anaerobe (*S. bovis*) predominated in ruminants fed an increased amount of concentrate during lactic acidosis (109). The activity of *S*. *bovis* only increases as a result of low pH (<5.75) conditions in the bovine rumen (113). According to McCaughern et al. (114), feeding a high-starch (220 g/kg of DM) diet to dairy cows could reduce rumen pH (0.15 units lower than normal pH) and increase milk yield (0.09 kg/d) and milk protein content (2.8 g/kg). Feeding a high-starch diet can directly affect the colonic lumen environment, which in turn alters the lumen-specific functional taxonomic groups (*Akkermansia*, unclassified *Christensenellaceae*, and *vadinBB60*). Consequently, the colonic epithelium makes a new niche that triggers cell apoptosis to achieve a functional transformation (98). These studies suggested that microbe–host interaction is vital for remodeling of hindgut homeostasis to allow adaptation to dietary perturbations.

Silage from various sources harbors different rumen microbial communities. Feeding of alfalfa silage increased the relative abundance of *F. succinogenes* and *R. flavefaciens* while reducing CH_4_ production in the cow rumen as compared to sweet sorghum silage. However, populations of *Ruminococcus albus* and *Ruminobacter amylophilus* showed no change (115). Contrarily, sheep fed an alfalfa hay diet had higher ruminal *Fibrobacter succinogenes* compared to *Ruminococcus* (116). According to Guo et al. (117), fermented corn stover showed a positive effect on ruminal bacterial diversity favoring four bacterial phyla; *Bacteroidetes, Lentisphaerae, Firmicutes*, and *Fibrobacteres*, which constituted 77% of total bacterial abundance. Additionally, feeding of fermented corn stover shifted the rumen fermentation kinetics in cows through increasing the relative abundance of *Prevotella* and stabilizing the rumen microbial ecosystem. Recently, it has been reported that feeding temperate grasses produce less enteric CH_4_ than tropical grasses in ruminants (118) as feeding low-quality tropical grasses emitted 17 g CH_4_/kg DM intake. However, a 10–25% decrease in CH_4_ production has been observed, when foliage and pods of trees and shrubs are included in the cattle diet (118).

A recent meta-analysis showed that feeding high-forage diets (>40% DM) reduced milk production (0.087 L/d) and milk lactose content (0.065 g/100 g) compared to high-concentrate diets (>40% DM) in sheep. However, fat content and conjugated linoleic acid concentrations were higher in the high-forage group (119). These findings convincingly reveal that the appropriate ratio of roughage and concentrate is required to optimize the rumen microbial ecosystem for better digestion and utilization of the dietary components while minimizing CH_4_ emission. Chemostatic feedback regulation (energy feedback), physical fill and feed passage rate of concentrate, and forage-type diets are involved in affecting the DM intake of ruminants. Moreover, inoculation of silage with specific groups of beneficial microbes can positively influence rumen fermentation kinetics and performance of animals on a sustainable basis.

### Interaction of Dietary Fat With Rumen Microbiome

Generally, supplementation of fatty acids (FA) is not required for microbial proliferation in the rumen because microbes can synthesize their own FA. Basal feed ingredients, including forages and grains, provide about 3–3.5% fat on a dry matter (DM) basis. However, for high-producing dairy cows, additional fat supplementation up to 2% of rumen-active fat (vegetable blends, oilseeds) and rumen-inert fat is usually recommended to make total dietary lipids up to 6–7% of DM (120). These dietary lipids are usually enriched in polyunsaturated fatty acids (PUFA) (121), which can make complexes with bacterial cell walls and are considered toxic to gram-positive bacteria in the rumen (122). In most ruminant diets, fat is below 5% of total DM. Higher dietary fat contents, primarily unsaturated FA, are discouraged owing to their adverse impacts on ruminal bacteria and feed degradation (123). However, rumen microbiota can detoxify unsaturated FA through the biohydrogenation process to reduce/eliminate the adverse effects on rumen fermentation (121, 124).

The addition of fats from plant or animal sources is an accepted approach for mitigation of CH_4_. However, consideration of fat supplementation to mitigate enteric CH_4_ emission depends on the cost and expected adverse effect on feed intake and digestibility (19). Improving the nutritive quality (high fat and fiber digestibility) of the offered diets has shown to reduce the DM intake in lambs but showed no effect on total tract digestibility of DM, organic matter (OM), crude protein, acid detergent fiber (ADF), and neutral detergent fiber (NDF) contents (125). Adding fat up to 6–7% of DM has shown no adverse effects on total tract digestibility (126). Recent studies have reported no effects of rumen-protected fats on NH_3_-N concentrations, total VFA, and overall bacterial population in sheep (127). Changes in rumen microbiota (*Acetitomaculum, Lachnospira*, and *Prevotella*) caused by an increased proportion of concentrates were considerably more significant than fat (128). *Fibrobacter* and *Ruminococcus* were most adversely affected among different bacterial genera, but such effects were highly variable for *Butyrivibrio* and *Prevotella*. These two genera (*Butyrivibrio* and *Prevotella*) include many species with diverse functions in metabolic pathways (129).

A decrease or no change was observed in major protozoa genera in response to the addition of linseed oil, particularly in high-concentrate diets (130). Moreover, increasing the degree of unsaturation reduces the protozoal count, but due to high random and animal variations, this change can be challenging to assess, which may explain inconsistent experimental data (131). Dietary supplementation of camelina oil has not shown any effect on ruminal protozoa (132), but a decrease in bacterial N and the number of cellulolytic bacteria was observed in diets supplemented with 8% dietary lipids (133). However, Bayat et al. (134) reported that the inclusion of camelina oil in the diet exhibited no effects on the relative abundance of protozoa, total bacteria, methanogens, fungi, and fiber-degrading bacteria.

Lipids from oilseeds, vegetable oils, and rumen-protected fat of vegetable oils are usually used as energy sources for dairy cattle (135). Oilseeds can be one of the efficient ways to reduce enteric CH_4_ production to mitigate CH_4_ emission from ruminants. Plant oils can mitigate CH_4_ by directly inhibiting rumen protozoa and methanogens and increasing the biohydrogenation of PUFA to act as a sink for hydrogen produced by rumen microbes (136). The utility of lipids to reduce enteric CH_4_ production is a better strategy as compared to antibiotics and ionophores like monensin. Several studies have reported adverse effects of FA, especially PUFA, on methanogenesis in the rumen (136). The anti-methanogenic effects of PUFA generally get intensified with the increase in double bond number per FA, as suggested by Czerkawski and Clapperton (137).

Supplementation of fat has been shown to reduce CH_4_ emission in ruminants consistently. However, various factors such as fat source, FA profile, basal diet, and fat type can affect the anti-methanogenic efficiency of dietary fats (93). This reduction in CH_4_ emission by dietary fat is mainly through the depressed fiber digestion in the rumen (138). However, according to McGeough et al. (125), a highly digestible fiber diet with higher fat content tended to increase CH_4_ emissions per kg of DMI and OMI, while the same amount of fat showed no effect on these parameters in a diet with low fiber digestibility. The inclusion of fat in a high-concentrate diet of sheep improved fat and conjugated linoleic acid contents of milk (119). Bayat et al. (139) reported a decrease in daily CH_4_ emission in lactating cows fed a low concentrate diet supplemented with sunflower oil.

Recently, moringa and camelina oils have shown to effectively reduce enteric *in vitro* CH_4_ production in different TMR through modulation of rumen microbes and shifting rumen kinetics (140, 140). Variable effects of vegetable oils and unsaturated FA on CH_4_ emission might be associated with the double bond number per FA, type of oil (free oil or whole seed), and composition (roughage-to-concentrate ratio) of the rations (134). An *in vitro* study of Vargas et al. (141) shows that supplementation of vegetable oil (sunflower and linseed) at 6% in high-concentrate TMR has the potential to reduce CH_4_ emission (up to 21–28%), butyrate concentration, and A:P, while increasing propionate concentration. Recently, a meta-analysis showed that addition of nitrates and vegetable oils in cattle diet has the ability to reduce CH_4_ emission up to 6–20% (118). Considering different studies regarding the reduction of CH_4_ emission, the addition of plant oils to ruminant rations is suggested as a feasible nutritional strategy with a cleaner repercussion on the environment.

## Dietary Manipulation of Rumen Function Using Natural Feed Additives

Dietary changes have been reported as a major factor that influences the dynamics of rumen microbial populations and resultant metabolic shifts leading to significant changes in ruminant production (142, 143). Many dietary interventions have been used in ruminants for the manipulation of the rumen microbiome to improve overall feed efficiency while reducing methanogenesis. A few dietary methodologies have been assessed for enhancing rumen fermentation, mainly to reduce CH_4_ emission. These strategies were focused to (i) improve feed efficiency using the quality feed, (ii) shift rumen fermentation pathways using assorted feed additives, and (iii) genetically manipulate host animals using selective breeding. Each strategy has some potential advantages and limitations. Recent issues of drug residues and antibiotic resistance have shifted the interest toward natural feed additives with potential abilities to modulate performance in ruminants. Many encouraging results have been observed by the application of different feed additives, including organic acids, probiotics, enzymes, and phytochemicals. Ideally, feed additives should diminish CH_4_ emission, enhance animals' energetic efficiency by increasing propionate concentration, improve N_2_ utilization efficiency by decreasing its excretion, optimize rumen pH, and improve fiber digestion (144).

Most important natural feed additives are phytochemicals produced by plants as secondary metabolites with diverse biological activities. Some potential effects of these feed additives and their mechanism of action as rumen modulators are described as below.

### Plant Secondary Metabolites as Rumen Modulator

Despite the sustainability of functional redundancy in the rumen microbiome, plant secondary metabolites have shown significant manipulation of the rumen microflora leading to a shift in fermentation dynamics and milk production in lactating animals (29, 31, 145–147). They have also shown to reduce the methanogenesis both *in vitro* and *in vivo* (29, 31, 76, 148, 149). Recently, numerous plant extracts have been investigated for their capacity to manipulate gut physiology and antimicrobial activity. Some plant metabolites, for example, saponins, tannins, and essential oils (EO), have shown promising potential for decreasing CH_4_ emission from animals. They have shown significant impacts on methanogens as well as protozoa, feed degradation/absorption, and fermentation parameters.

#### Effect of Saponins on Rumen Methanogenesis and Fermentation Characteristics

Saponins are a class of plant secondary compounds with diverse chemical compositions and biological activities (31). Saponins comprise mainly sapogenins and glycosides found mostly in angiosperms. Steroidal and triterpenoid saponins are two significant groups of saponins, which protect plants from bacterial and fungal invasions (150). Saponin-rich plants such as lucerne and soybeans are broadly utilized for ruminant feeding. Additionally, *Quillaja saponaria* (soapbark), *Yucca shidigera* (yucca), and *Sapindus s*p. (soap berries) are considered as well-known sources of saponins (151). Saponins have a fat-soluble nucleus, and they have shown antibacterial, antitumor, and anti-inflammatory properties in animals (152, 153). Saponins mediate rumen fermentation mainly by decreasing protein degradation and concentrations of urea and NH_3_ in the rumen, leading to an increased flow of amino acids to the small intestine. Potential effects of saponins are associated with N_2_ metabolism, mainly through their lethal effect on protozoa, which are primarily responsible for proteolytic activity in the rumen (154). Saponins reduce the protozoal population and some methanogens associated with protozoa, although their effect on methanogens does not always correlate with the effect on protozoa (31). The interaction of sterol moiety with saponin, present in the protozoa membrane, has an association with the antiprotozoal effect of saponins (154).

It is generally expected that a reduction in the population of methanogens can decrease CH_4_ emissions. Extracts of *S. sesban* have shown to decrease protozoal and methanogen populations but surprisingly did not decrease CH_4_ production (155). There was a frail relationship between methanogenesis and methanogens in the rumen. This is mainly because the enhanced expression of some methanogenic genes may lead to enhanced methanogenesis that ultimately compensated a decrease in the overall number of methanogens (156). These findings provide explicit insight regarding, managing gene interactions from the microbiome to enhance nutrient utilization efficiency.

Tea saponins have been extensively used in many studies *in vitro* and *in vivo* to evaluate the effects on rumen fermentation and methanogenesis. Variable results regarding microbial population and rumen fermentation parameters have been observed in response to the supplementation of different sources of saponins (Tables 1, 2). Despite the decline observed in methanogenesis through a direct decrease in protozoa populations under *in vitro* conditions, the same plant extract (0.52% on DM basis) failed to reduce daily CH_4_ production in lactating dairy cows (162). This suggests that the impacts of tea saponins under *in vitro* conditions must be confirmed *in vivo* to develop effective CH_4_-mitigating strategies.

**Table 1:** Effect of saponins on rumen microbial population.

**Sources**	**Test system/dose**	**Diet**	**Total bacteria**	**Protozoa**	**Methanogens**	***F.S***	***R.F***	***R.A***	***B.F***	**References**
Tea saponin	*In vivo* or *in vitro* both in ewe 3 g/d	TMR + wildrye hay	=	↓	=	↑	=	=	=	(157)
Tea saponin (Lerak) Tea saponin (Hibiscus)	*In vitro* rumen fluid from Cattle (2 and 4%)	Cassava leaf silage	= =	= =	= =	= =	= =	= =	NF NF	(158)
Quillaja saponin	*In vitro* rumen cows (0.6 g/L)	TMR	↑	NF	=	NF	NF	NF	NF	(159)
Combination (Enterolobium cyclocarpum and Gliricidia sepium)	*In vivo* or *in vitro* both in heifer	TMR 3.3% of 15% DM	=	=	=	NF	NF	NF	NF	(160)
Tea saponin	*In vitro* rumen fluid from cows 0.77%	F:C (50:50)	=	=	=	NF	NF	NF	NF	(161)
Tea saponin	Chambers 0.52% *in vitro* bottles	TMR	NF	↓	NF	NF	NF	NF	NF	(162)
Quillaja saponin	Open chambers (0.6 g/L)	F:C (50:50)	=	↓	↓	=	↑	↑	NF	(163)

*F.S, Fibrobacter succinogenes; R.F, Ruminococcus flavefaciens; R.A, Ruminococcus albus; B.F, Butyrivibrio fibrisolvens; NF, not found; ↑, increase; ↓, decrease; =, no effect*.

**Table 2:** Effects of saponin on methanogenesis, rumen fermentation, and feed degradability.

**Sources**	**Test system/dose**	**Diet**	**CH_**4**_**	**NH_**3**_**	**tVFA**	**DMI**	**Acetate**	**Butyrate**	**Isobutyrate**	**Propionate**	**Isovalerate**	**Valerate**	**Acetate/** **Propionate**	**DMD**	**References**
Tea saponin	*In vivo* or *in vitro* both in ewe 3 g/d	TMR + wildrye hay	=	↓	↑	=	=	↑	↑	↑	↑	=	↓	↑	(157)
Tea saponin (Lerak) Tea saponin (Hibiscus)	*In vitro* rumen fluid from cattle (2 and 4%)	Cassava leaf silage	= ↑	↓ ↓	= =	NF NF	↑ ↑	↑ =	= =	↑ ↓	= =	↑ =	NF NF	↑ ↑	(158)
Quillaja saponin	*In vitro* rumen fluid from cows (0.6 g/L)	TMR	=	↓	=	NF	=	=	=	=	=	=	=	=	(159)
Combination of kulthi, patha, and aritha	*In vitro* rumen fluid from male buffaloes 2%	F:C (80:20)	↓	NF	↓	NF	=	↓	=	=	=	=	↓	↑	(164)
Combination of Enterolobium cyclocarpum and Gliricidia sepium	*In vivo* or *in vitro* both in heifer	TMR 3.3% of 15% DM	=	NF	=	=	=	=	=	=	NF	NF	=	=	(160)
Sapindus mukorossi fruits acetone extract	Buffalo rumen 125 ml bottles fitted 0.5 ml	Oat hay	↓	=	NF		=	=	=	NF	NF	NF	=	=	(165)
Alfalfa saponins	*In vivo* lamb 0.4%	F:C (50:50)	NF	NF	NF	=	NF	NF	NF	NF	NF	NF	NF	↑	(166)
Tea saponin	Open chambers 0.52% *in vitro* bottles	TMR	↑	=	=	↓	=	=	=	NF	NF	NF	=	=	(162)

*F:C, forage to concentrate ratio; TMR, total mixed ration; tVFA, total volatile fatty acid; DMI, dry matter intake; DMD, dry matter degradability; NF, not found; ↑, increase; ↓, decrease; =, no effect*.

Inactivation of saponins has been observed through deglycosylation into sapogenins by the rumen microorganisms that lead to the transitory antiprotozoal property of saponins. There are two approaches to improve the effectiveness of saponins and reduce their degradation by rumen microbes. One possible method is to use a combination of saponins with glycosidase-inhibiting iminosugars (167). The second option is altering the saponin structure, such as by combining ivy saponins with stevia extract. Hederagenin bis-succinate (HBS) obtained by hydrolysis of ivy fruit extract has shown to shift fermentation toward propionate, attributed to its structural modifications that mediated the diversity of bacterial communities (167).

Recently, tea saponins have been supplemented in alfalfa hay and soybean hull-based fiber diets and exhibited their ability to alter ruminal lipid metabolism in cattle through reducing the relative abundance of *Lachnospiraceae* (168). Furthermore, tea saponins have also been shown to effectively decrease N_2_ emission in sheep (157). However, studies have revealed that the activity of saponins fluctuates and even reduces during long-term studies (158), probably because of microbial adaptation (169). Moreover, saponins can increase the propionate ratio at the expense of both acetate and butyrate (158). Studies have shown that a combination of garlic oil, nitrate, and saponins can additively lower CH_4_ emission with similar rumen fermentation and degradability (163). Archaeal growth was inhibited by all treatments, but the abundance of *F. succinogenes R. albus*, and *R. flavefaciens* varied. According to Liu et al. (157), tea saponin did not affect methanogens and the total bacterial population including *R. flavefaciens R. albus*, and *Butyrivibrio*. However, protozoa were effectively reduced in response to tea saponins.

All saponins have no inherent antiprotozoal activity; that is why their biological activity can be affected by even small changes in their structure. For instance, sapogenins like asiatic acid and madecassic acid have more ability for the inhibition of protozoa than their corresponding saponins (Re and Rh_1_ and madecassoside). Therefore, further research is warranted to understand the deglycosylation of saponins and the nature of their antiprotozoal activity to devise effective ways to use saponins for CH_4_ mitigation in ruminants (170).

#### Effect of Tannins on Rumen Methanogenesis and Fermentation Characteristics

Tannins are polyphenolic compounds with molecular weights ranging from 500 to 5,000 Da with two major groups [i.e., condensed tannins (CT) and hydrolyzable tannins (HT)]. Tannins can bind with dietary proteins, starch, and sugar by making strong complexes at pH 3.5–7 (150). Tannins are widely distributed in different plant species, particularly in cereals, legumes, and fruits. They can limit the digestibility and nutritional value of plants considerably when their concentration reaches more than 5% (171). The action of tannins in the rumen is not entirely well-conceived yet (172). Although they possess bacteriostatic effects, the association of tannins with the rumen microbes is different, as hydrolyzable tannin is more susceptible to microbial hydrolysis than condensed tannin (173). They can limit the degree of microbial hydrolysis along with direct inhibition of methanogens. Additionally, they can also lower methanogenesis indirectly by decreasing H_2_ availability by reducing fiber digestion (Figure 3). Tannins can modify the ruminal microbiome, reduce protein degradation, decrease methanogenesis, and inhibit FA biohydrogenation (174, 175).

Variable results regarding the shifting of microbial population and rumen fermentation parameters have been observed in response to the supplementation of different sources of tannins (Tables 3, 4). Studies have reported quite different effects of tannin supplementations regarding CH_4_ mitigation. Some studies have also shown that tannins indirectly impede the degradation of fiber (76). A recent study showed that supplementation of acacia tannin (15 g/d/animal) reduced short-chain fatty acids (SCFA) and acetate (molar percentage) in lambs affected by gastrointestinal nematode infection. Furthermore, supplementation also increased the diversity and abundance of butyrate-producing and other beneficial bacteria (including probiotic species like *Bifidobacterium* and *Lactobacillusamino*), while enhancing the amino acid metabolic pathways and purine, pyrimidine, and sphingolipid metabolism (176). However, the precise mechanism of action and extent of contributory effects of acacia tannin on the ruminal microbiome are still not clear. Supplementation of tannic acid has shown to reduce the CP digestibility and CH_4_ production in beef cattle (198). Dietary supplementation of HT (chestnut) and CT (quebracho) increased the relative abundance of *Butyrivibrio fibrisolvens* by 3 and 5-fold, respectively, in the rumen of dairy sheep. On the other hand, they decreased the *B. proteoclasticus* population by 5 and 15-fold, respectively (199). Inhibition of rumen bacteria by CT is probably due to interactions between CT present in the tannin structure and the specific substrate (e.g., protein, bacterial cell walls, etc.) to which it binds (200). The addition of CT extracts to the diet reduced populations of methanogenic archaea and some cellulolytic bacteria (*R. flavefaciens*) (201). These reports suggest that dietary sources of HT and CT can affect the rumen microbiome quite differently owing to their structural variations.

**Table 3:** Effect of tannins on rumen microbial population.

**Sources**	**Test system/dose**	**Diet**	**Total bacteria**	**Protozoa**	**Methanogens**	***PV***	***RC***	***RB***	***BV***	**References**
Acacia mearnsii	*In vivo* lamb 15 g daily dose	TMR	NF	NF	NF	↓	=	NF	NF	(176)
Chestnut tannin extract	*In vitro* Ewe rumen 16 g/kg DM of CHT extract	TMR	=	=	=	=	=	=	=	(177)
Tannic acid	*In vivo* cattle 16.9 g TA/kg DM	(TMR) Low CP High CP	= =	NF	NF	= =	= =	NF	= =	(178)
DFPP condensed tannin (6.9%)	*In vitro* steers DFPP levels 1% 2% 3% 4%	F:C (30:70)	NF	↓ ↓ ↓ ↓	NF	NF	NF	NF	NF	(179)
HT Chestnut Tannic acid Gallic acid	Beef cattle 2% 1.5% 1.5%	Alfalfa silage	NF	= = =	NF	NF	NF	NF	NF	(180)
Tannin extracted from pomegranate peel	Lambs 29% 25% 30%	Recycled poultry bedding	= ↓ ↓	NF	NF	NF	↓ ↓ ↓	NF	NF	(181)
HT Gallic acid	*In vitro* 0.5% 1% 2%	TMR	NF	= = =	NF	NF	NF	NF	NF	(182)
HT Syzygium cumini CT Machilus bombycina	*In vivo* Lambs 14.08 and 4.29 g/kg DM	F:C (50:50)	= ↑	↓ =	NF	NF	NF	NF	NF	(183)
Tannin from chestnut, valonea, sumac and grape seed	*In vitro* Non-lactating cows 1.5 g/d	TMR	NF	NF	=	NF	↓	NF	NF	(184)
i) HT chestnut, tara ii) CT mimosa, gambier	*In vivo* lamb 40 g/kg commercial extract	Concentrate	= = = =	= ↓ = ↓	= = ↓ ↓	↓ = = ↓	= = = =	= = ↓ =	↓ = = ↓	(185)
HT chestnut CT mimosa	*In vivo* sheep 10%	Grass hay	NF	NF	NF	= =	↑ ↓	= =	↑ ↓	(186)

*PV, Prevotella; RC, Ruminococcus; RB, Ruminobacter; BV, Butyrivibrio; NF, not found; ↑, increase; ↓, decrease; =, no effect*.

**Table 4:** Effects of tannins on methanogenesis, rumen fermentation, and feed degradability.

**Sources**	**Test system/Dose**	**Diet**	**CH_**4**_**	**NH_**3**_**	**tVFA**	**DMI**	**Acetate**	**Butyrate**	**Isobutyrate**	**Propionate**	**Isovalerate**	**Valerate**	**Acetate/** **Propionate**	**DMD**	**References**
Tannin-containing hay	*In vivo* cows and heifers	Hay	↓	NF	NF	NF	NF	NF	NF	NF	NF	NF	NF	NF	(187)
ATE	*In vivo lambs* 42 g/kg DM	Urea-containing diet	=	↓	=	=	↓	=	NF	↑	NF	↑	↓	↓	(188)
Acacia mearnsii	*In vivo* lamb 15 g daily dose	TMR	NF	=	NF	NF	↓	↑	=	=	NF	↑	=	NF	(176)
lipid encapsulated- ATE	*In vitro* 24 h	TMR	↓	↓	NF	NF	NF	NF	NF	NF	NF	NF	NF	NF	(189)
Oak tannin extract	*In vivo* lactating cows 169 g/DM	TMR including linseed	=	NF	NF	=	NF	NF	NF	NF	NF	NF	NF	=	(190)
DFPP condensed tannin (6.9%)	*In vitro* steers DFPP levels 1% 2% 3% 4%	F:C (30:70)	↓ ↓ ↓ ↓	= = = =	= = = =		↓ ↓ ↓ ↓	= = = =	NF	↑ ↑ ↑ ↑	NF	NF	↓ ↓ ↓ ↓	↑ ↑ ↑ ↑	(179)
i) HT chestnut, tara ii) CT mimosa, gambier	*In vivo* lamb 40 g/kg commercial extract	Commercial concentrate diet	NF	= = = =	= = = =	NF	= = = =	= = = ↓	↓ = = =	= = = =	↓ = = =	= = = =	= = = =	NF	(185)
Quebracho tannin extract	Crossbred heifers, 1% 2% 3% 4%	Low-quality tropical *Pennisetum purpureum* grass	= = ↓ ↓	= = = =	= = = =	= = = ↓	= = = =	= = = =	= = = =	= = ↑ ↑	= = = ↓	= = = =	= = ↓ ↓	= = = ↓	(191)
40% distillers grains and solubles with CT	Cannulated crossbred beef heifers 2.5% CT extract	High protein finishing diets	↓	=	↓	=	=	↑	=	↓	=	=	↑	↓	(192)
Tannic acid	*In vivo* cattle 16.9g TA/kg	(TMR) Low CP High CP	NF	↓ ↓	↓ ↓	= =	↑ ↑	= =	= =	= =	↓ ↓	↓ ↓	↑ ↑	↓ ↓	(178)
HT Chestnut tannic acid gallic acid	Beef cattle 2% 1.5% 1.5%	Alfalfa silage	= = ↓	= ↓ =	↑ = ↑	= = =	= = =	= = =	↑ ↑ ↑	= = =	= = =	= = =	= = =	= = =	(180)
HT syzygium cumini CT Machilus bombycina	*In vivo* lambs 14.08 and 4.29 g/kg DM	F:C (50:50)	↓ ↓	↑ ↓	↓ ↓	= =	↓ ↓	↓ =	= =	↓ ↓	NF	↓ ↓	↓ ↓		(183)
HT gallic acid	*In vitro* 0.5% 1% 2%	TMR	= = =	= = =	= = =	= = =	= = =	= = =	= = =	= = =	↓ ↓ ↓	= = =	= = =	NF	(182)
CT Cistus ladanifer	*In situ* ram CT levels 4% 8% 12%	Lucerne silage	NF	↓ ↓ ↓	NF	NF	NF	NF	NF	NF	NF	NF	NF	↓ ↓ ↓	(193)
Tannin extracted from pomegranate peel	Lambs 29% 25% 30%	Recycled poultry bedding	NF	= ↓ ↓	= = =		= = =	= = =	NF	= = =	= = =	= = =	= = =	NF	(181)
Combination of TA and AF	*In vitro* TA (0.02 g) + AF (0.02 g) + Wheat barn (0.01 g)	Commercial concentrate diet	↓	NF	=	NF	=	NF	NF	=	NF	NF	NF	=	(194)
CT	*In-vitro* incubation 2.5% 5% 7.5%	Cassava silage	NF	= = =	NF	↑ ↑ ↑	↑ ↑ ↑	= = =	NF	= = =	NF	NF	NF	= = =	(195)
HT Acacia nilotica	*In vitro* Sheep 25% 50% 75% 100%	Acacia nilotica leaves	↑ ↓ ↓ ↓	NF	= ↓ ↓ ↓	NF	↑ ↑ ↑ ↑	↓ ↓ ↓ ↓	↑ ↓ ↓ ↓	↑ ↓ ↓ ↓	↑ ↑ ↓ ↓	↓ ↓ ↑ ↑	↑ ↑ ↑ ↑	NF	(196)
Chestnut tannin, glycerol	*In vitro* Bull Chestnut, 30% Glycerol, 30% Both 60%	Ensiled cassava leaves F:C (60:40)	↑ ↓ =	↓ ↑ ↓	= = =	NF	= = =	= ↑ =	= ↑ =	= = =	↑ = ↑	= = =	= = =	= = =	(197)
HT Chestnut CT mimosa	*In vivo* sheep 10%	Oil diets	NF	NF	↑ ↓	↑ ↓	= =	= =	↑ ↓	= =	↑ ↓	= =	↑ ↓	NF	(186)

*F:C, forage to concentrate ratio; TMR, total mixed ration; tVFA, total volatile fatty acid; DMI, dry matter intake; DMD, dry matter degradability; NF, not found; ↑, increase; ↓, decrease; =, no effect; HT, hydrolysable tannins; CT, hydrolysable tannins; DFPP, dragon fruit peel powder; TA, tannic acid; AF, Allium fistulosum L.; ATE, acacia tannin extract*.

Different sources of tannin have been used to mitigate CH_4_ emission while increasing animal performance. Gallic acid is a phenolic monomer and one of the ruminal decomposed metabolites of tannic acid (173), and it serves as an essential bioactive component in modifying the rumen fermentation. As a subunit of HT, gallic acid has the potential to decrease the environmental impact of ruminants (by lowering CH_4_ and NH_3_ emissions) without decreasing animal performance (180). Gallic acid (0.015%) decreased the urine nitrogen emissions by 28.5% (CP 0.11% DM) and 30.9% (CP 0.15% DM) when applied to the soil (202). A recent study showed that gallic acid can inhibit undesirable microorganisms such as *Clostridium, Listeria*, and *Escherichia coli* during ensiling and can improve fermentation quality and protein preservation (203). Extracts of HT (tara) and CT (mimosa and gambier) inhibited the activity of methanogens and protozoa without affecting ruminal fermentation and animal production (185). Condensed tannins have shown better protein efficiency and growth rate of lambs as it can protect dietary proteins (e.g., soybean meal) from ruminal degradation, leading to reduction in digestive losses (204). Recently an *in vitro* study of Saminathan et al. (205) showed that tropical legumes having CT with different molecular weights can serve as potential feed additives to mitigate CH_4_ production with no adverse effects on rumen fungal microflora and fiber digestion. Contrarily, Rira et al. (196) reported that *in vitro* HT (*A. nilotica*) are more promising for suppressing methanogenesis than CT (from *C*. *calothyrsus* and *L*. *leucocephala*). Chestnut tannin possesses sufficient potential to reduce methanogenesis, without compromising feed efficiency and animal performance due to its neutral effect on NDF digestibility (177). According to Witzig et al. (184), CH_4_ emission was reduced in response to monensin and chestnut tannin supplementation, owing to the lower abundances of *M. ruminantium* and *M. stadtmanae*.

Tannic acid (0–1.25 mg/mL) can alter microbial activities and improve feed efficiency in ruminants. However, the increased tannic acid concentration may lead to the complete inhibition of ruminal bacteria in sheep (206). Mimosa CT could reduce the abundance of specialized fibrolytic bacteria and inhibit the biohydrogenation process as compared to chestnut HT. Further investigations are required to evaluate the impact of different sources of tannins on ruminal biohydrogenation (186). Hydrolyzable tannins are considered more suitable for CH_4_ mitigation than CT. *A. nilotica* (HT) showed a more potent inhibitory effect on CH_4_ production compared to *C*. *calothyrsus* and *L*. *leucocephala* (CT). It may be attributed to the fact that HT (e.g., gallic acid subunits) directly inhibit methanogens, but the action of CT on rumen CH_4_ production is variable (172, 207). However, long-term trials are required to assess the possible adaptation of rumen microbes toward the optimal level of HT and its subunit, gallic acid, to avoid their adverse effects on animal performance (196).

Tannin containing hay has been shown to reduce the CH_4_ emission (5.4 DM vs. 3.5 ml/g) and urea N excretion in beef cattle (187). To reduce the adverse effect of tannin on DM intake, the encapsulation of tannin extract could be considered as a better strategy as the slow release of tannin also improves its utilization (208). Recently, Adejoro et al. (189) determined the effect of crude (40 g/kg feed) and lipid encapsulated-acacia tannin (50 g/kg feed) extracts on sheep fed TMR. They reported a 30% and 19% reduction in CH_4_ production (g/kg DM) with crude and encapsulated-acacia tannin, respectively. However, crude tannin also imparted an adverse effect on NDF digestibility compared to encapsulated-acacia tannin. Supplementation of tannin could reduce the NH_3_ toxification which is usually produced in response to NPN addition in ruminant diets (208). More recently, Adejoro et al. (188) reported that supplementation of 42 g acacia tannin /kg feed DM did not reduce CH_4_ production in lamb fed nitrate or urea as an NPN source. A possible reason is the comparatively higher affinity of acacia tannin for feed protein than microbial protein or microbial enzymes (209). Tannin has the potential to reduce the excretion of a more volatile form of N into the environment by decreasing rumen degradability of CP and shifting N excretion from urine to feces (210). A meta-analysis showed reduction in ruminal ammonia N (16%), milk urea (9%), and urinary N excretion (11%) in response to supplementation of tannin in lactating dairy cows. However, tannin exhibited no effect on fat- and protein-corrected milk yield (211). A short-term effect of *A. mearnsii* (30 g/kg) showed a negative effect on CH_4_ production in dairy cows.

Dietary supplementation of oak tannin has also been shown to reduce the urinary N excretion by 12% while increasing α-linolenic acid content in milk by 17.7% without affecting CH_4_ production (190). Such divergent findings may possibly be due to different dietary concentrations of tannin and a variable number of hydroxyl groups in their structure (207). Studies have suggested an association of milk FA profile with CH_4_ emission, which can assist in determining the impact of tanniferous supplement against enteric CH_4_ production (212). The medium-chain FA (lauric and myristic acids) have shown a positive correlation with enteric CH_4_ production as these FA are synthesized (*de novo* synthesis) in the mammary gland from ruminal acetate and butyrate (213). In this regard, it has been reported that supplementation of quebracho tannins (30 g/kg DM) could reduce myristic acid content in dairy cows, which reveals the negative effects of tannin on fiber digestibility (214). However, long-chain FA (pentadecanoic and heptadecanoic acid) exhibited a negative correlation with CH_4_ emission as their *de novo* synthesis is mediated from ruminal propionate (215). However, further careful investigations are warranted to corroborate this relationship.

Tannins also possess antioxidant properties as they can scavenge free radicals due to hydroxyl groups, degree of polymerization, and redox activities (216–218). Supplementation of tannin has been shown to improve the antioxidant status of cattle and sheep (219–221). Hydrolyzable tannins are considered the most potent antioxidants, which can prevent cellular damage and neutralize free radicals (222), while condensed tannins (catechin) also possess antioxidant activities (223). Pomegranate as hydrolyzable tannin has shown better antioxidant activity as tested on cultured bovine aortic endothelial cells; it has no adverse effects on cell viability and apoptosis. Pomegranate has also exhibited protective effects against membrane lipid peroxidation, owing to its potent ability to reduce the production of intracellular reactive oxygen species (224). However, the optimum level of tannin for antioxidant capacity and its putative mechanism of action in animal tissues require further elucidation. A recent study has revealed that rumen microbial taxa (*Bifidobacterium, Lactobacillus*, and *Schwartzia*) exhibited a strong association with host antioxidant capacity and immunomodulatory functions (225).

Prolonged use of the purified form of secondary compounds can lead to antimicrobial resistance; however, tannin supplementation as a crude extract of mixtures (having different molecular sizes) offers a major advantage to control antimicrobial resistance (226). Moreover, studies have shown that CT-rich diets can effectively decrease CH_4_ emissions per unit of DMI over a range of dietary CP from 15 to 25%. For example, a decrease up to 25 to 50% was observed in *in vitro* CH_4_ production in steers grazing on winter wheat forage (15 to 18% CP) supplemented with quebracho CT extract at 10–20 g/kg DMI (227, 228). This shows the effective inhibitory effects of tannins on ruminal protein degradation and CH_4_ emission but requires careful selection of diets and nutrient composition to avoid adverse effects on feed digestibility and efficiency (229). However, further studies are required to fully understand the mechanism of action of tannins regarding modulation of the rumen microbiome, potential inhibitory effects on methanogens and protozoa, and their optimum inclusion levels to elucidate their potential for CH_4_ mitigation. Furthermore, focused investigations are required to explore the optimum levels and types of tannins and feeding conditions to reduce GHG emission in commercial ruminant production systems.

#### Effect of Essential Oils on Rumen Methanogenesis and Fermentation Characteristics

Essential oils are terpenoids (monoterpenoids and sesquiterpenoids) and phenylpropanoid compounds with characteristic flavors and odors, formed by different plants (herbs and spices). They contain numerous chemical substances, for example, alcohols, hydrocarbons, ketones, aldehydes, ethers, and esters, and mostly EO are lipophilic complexes (230, 231). Various studies have been performed to evaluate the effect of EO on rumen fermentation and feed degradability. Many *in vitro* and *in vivo* trials have proved the favorable effect of EO in reducing CH_4_ production and altering microbial populations (Tables 5, 6). The potential effect of EO on rumen fermentation and methanogenesis is mainly mediated by their antimicrobial activities owing to their interaction with cell membranes of microbes (by disrupting membrane stability of lipid bilayer). They are most effective against gram +ve bacteria and possess almost no activity against gram –ve (because of their hydrophilic bilayer) except thymol and carvacrol (232). Garlic oil has shown inhibition of HMG-CoA reductase, leading to membrane instability and, eventually, cell death in methanogenic archaea. Recently, metagenomic analysis of goat rumen revealed that EO cobalt complexes significantly manipulated the structural and functional profile of rumen microbiota. It was revealed that *Bacteroides* sp. and *Succinivibrio* sp. showed a positive correlation with enhanced VFA production in supplemented groups. Moreover, functional prediction pathway analysis exhibited upregulation of lipid and carbohydrate pathways by EO (233).

**Table 5:** Effect of various EO and their compounds on rumen microbial population.

**Sources**	**Test system/dose**	**Diet**	**Total bacteria**	**Protozoa**	**Methanogens**	***F.S***	***R.F***	***R.A***	***B.F***	**References**
Oregano essential oil	*In vitro* (13, 52, 91, and 130 mg/L	F:C (65.5:34.5)	=	NF	NF	↓	=	=	=	(239)
Oregano oil and carvacrol	Cannulated cows (50 mg/kg of DM)	TMR	NF	=	NF	NF	NF	NF	NF	(240)
Oregano essential oil	*In vivo* sheep 4 g/d 7 g/d	F:C (65.5:34.5)	↑ =	↓ ↓	NF	↑ =	↑ ↓	↑ =	NF	(241)
Essential oil-cobalt	Goat 52 mg/d 91 mg/d	Concentrate	↓ ↓	NF	= =	↑ ↓	↓ =	↓ =	↓ =	(233)
Plant-derived EO (carvacrol, eugenol and thymol)	*In vitro* and *vivo* both Control LCP LCP 35 g/d	TMR	NF	= = =	NF	NF	NF	NF	NF	(242)
Mixture of cinnamaldehyde, thymol, and eugenol	Heifer 1 g/kg substrate 2 g/kg substrate	F:C (60:50) 24 h	NF	= ↑	NF	= =	= ↑	↑ =	= ↑	(243)
Thymol:carvacrol	*In vitro* 0:100, 20:80, 40:60, 60:40, 80:20, 100:0	Rumen culture of bovine	= ↑ ↓ ↑ ↑ ↑	= ↑ ↓ ↑ ↑ ↑	NF	NF	NF	NF	NF	(244)
Java cardamom	*In vitro* cow 25 mg/l 50 mg/l 75 mg/l 100 mg/l	F:C (60:40)	NF	= = = =	NF	NF	NF	NF	NF	(245)
Blend of cinnamaldehyde and garlic oil	*In vitro* 0.0043% of DM	F:C (50:50)	NF	=	NF	NF	NF	NF	NF	(246)
Anise EO Anise extract	*In vitro* rumen buffer 250 μL /30 ml 500 μL /30 ml 750 μL /30 ml 1,000 μL /30 ml 250 μL /30 ml 500 μL /30 ml 750 μL /30 ml 1,000 μL /30 ml	F:C (40:60)	NF	= = = = = = = =	NF	NF	NF	NF	NF	(247)

*F:C., forage to concentrate ratio; TMR, total mixed ration; NF, not found; ↑, increase; ↓, decrease; =, no effect; EO, essential oils; F.S, Fibrobacter succinogenes; R.F, Ruminococcus flavefaciens; R.A, Ruminococcus albus; B.F, Butyrivibrio fibrisolvens; LCP, low CP diet*.

**Table 6:** Effects of various EO and their compounds on methanogenesis, rumen fermentation, and feed degradability.

**Sources**	**Test system/dose**	**Diet**	**CH_**4**_**	**NH_**3**_**	**tVFA**	**DMI**	**Acetate**	**Butyrate**	**Isobutyrate**	**Propionate**	**Isovalerate**	**Valerate**	**Acetate/** **Propionate**	**DMD**	**References**
Oregano essential oil	*In vitro* (13, 52, 91, and 130 mg/L	F:C (65.5:34.5)	↓	↓	↓	NF	↓	↓	↓	↓	↓	↓	↑	↑	(239 )
Oregano oil and carvacrol	Cannulated cows (50 mg/kg of DM)	TMR	=	=	=	=	=	=	NF	↑	NF	=	↓	=	(240 )
Dried oregano	Dairy cows (18, 36, and 53 g DM/kg of dietary DM in low EO	TMR	=	=	=	=	=	=	NF	=	NF	=	=	=	(248 )
Essential oil-cobalt	Goat 52 mg/d 91 mg/d	Concentrate	NF	↓ ↓	↑ =	= =	↑ =	= =	NF	= =	NF	NF	NF	NF	(233 )
Lippia turbinate Tagetes minuta Mix	*In vitro* sheep 1 ml in fermenter daily	F:C (80:20)	↓ ↓ ↓	= ↓ ↓	= = =	NF	= = =	= = =	= = =	= = =	= = =	= = =	= = =	↓ ↓ ↓	(249 )
Cashew and Castor	*In vitro* cow 1 g/d 2 g/d 4 g/d 8 g/d	F:C (20:80)	NF	= = = =		= = = =	= = = =	= = = =	NF	= = = =	NF	NF	= = = =	= = = =	(250 )
Mixture of cinnamaldehyde, thymol, and eugenol	Heifer 1 g/kg substrate 2 g/kg substrate	F:C (60:50) 24 h	NF	= ↑	= =		= =	= =	NF	= =	NF	NF	= =	↑ =	(243 )
Thyme Mint Savory	*In vitro* cow 50 μl/l of total culture medium	TMR	NF	↓ ↓ ↓	NF	NF	NF	NF	NF	NF	NF	NF	NF	↑ ↑ ↑	(251 )
Lavandula angustifolia Santalum spicatum	Sheep μl/g DMI 62.5 125 250 500 62.5 125 250 500	Hig- concentrate diet	↑ = ↓ ↓ ↓ ↓ ↓ ↓	↓ ↓ ↓ ↓ ↓ ↓ ↓ ↓	↓ ↓ ↓ ↓ = = = =	NF	= = ↑ = = = = =	= ↑ = ↓ = = = =	NF	= ↓ ↓ ↓ ↑ ↑ ↑ ↑	NF	NF	= ↑ ↑ ↑ ↓ ↓ ↓ ↓	= = ↓ ↓ = = = ↓	(252 )
Thymol:carvacrol ratio	*In vitro* 0:100 20:80, 40:60, 60:40, 80:20, 100:0	Rumen culture of bovine	= = = = = =	= ↑ ↓ ↑ ↑ ↑	NF	NF	NF	NF	NF	NF	NF	NF	NF	= = = = = =	(244 )
EO	Dairy cow 1 g/d	TMR	↓	NF	NF	↑	NF	NF	NF	NF	NF	NF	NF	NF	(253 )
Blend of EO (cresols, thymol, limonene, vanillin, guaiacol, eugenol, and salicylate)	*In vitro* cow 20 ml/l 100 ml/l 200 ml/l 600 ml/l 1,000 ml/l	F:C (60:40)	= = = ↓ ↓	= = = = ↓	= = = ↓ ↓	NF	= = = = ↓	= = = ↑ ↑	= = = ↓ ↓	= = = ↓ ↓	= = = = ↓	= = = ↓ ↓	= = = = ↑	= ↓ = ↓ ↓	(254 )
Lemon grass EO	Lamb, 1 ml/kg of DM	F:C (15:85)	NF	NF	=	=	=	↓	↓	=	↓	↑	NF	=	(255 )
Java cardamom	*In vitro* cow 25 mg/l 50 mg/l 75 mg/l 100 mg/l	F:C (60:40)	= = = =	= = ↓ =	= = = =	NF	= = = =	= = = =	NF	= = = =	NF	NF	= = = =	↓ = = =	(245 )
Plant-derived EO (carvacrol, eugenol and thymol)	*In vitro* and in vivo both Control LCP LCP 35 g/d	TMR	NF	= = =	= = =	↑ ↓ ↓	= = =	= = =	= = =	= = =	= = =	= ↓ =	= = =	= = =	(242 )
Blend of cinnamaldehyde and garlic oil	Sheep 0.0043% of DM	F:C (50:50)	=	=	=	=	=	=	=	=	↑	=	=	=	(246 )
Microencapsulated blend of EO	Sheep 0.02% 0.04%	TMR	↓ ↓	= =	↑ ↑	= =	= =	↑ ↑	= =	= ↑	= =	= =	= ↓	= =	(237 )
Citrus essential oils	*In vitro* 0.8 mL/L rumen volume	TMR (3 weeks)	=	↓	↓	=	↓	=	NF	=	NF	NF	=	=	(256 )

*F:C, forage to concentrate ratio; TMR, total mixed ration; tVFA, total volatile fatty acid; DMD, dry matter degradability; DMI, dry matter intake; NF, not found; ↑, increase; ↓, decrease; =, no effect; EO, essential oils; LCP, low CP diet*.

Some studies have also reported a few unfavorable effects of using EO as feed additives as they depressed synthesis of VFA by reducing feed degradability (234). These harmful effects might be due to their extensive and non-specific antimicrobial properties in the rumen. In a study, no effect on the rumen microbiome has been observed by supplementation of a blend of EO having thymol, guaiacol, eugenol, vanillin, salicylaldehyde, and limonene (235). The desirable effects of EO on the rumen physiology are mainly attributed to their phenolic compounds, which possess the potent ability to affect the activity of both gram-positive and gram-negative bacteria (236). Inhibition of gram-positive bacteria in the rumen can potentially increase the propionate concentration (235). Supplementing a blend of EO (cinnamaldehyde, eugenol, carvacrol, and capsicum oleoresin) firstly increased rumen acetate concentration, which was replaced by propionate concentration afterward. This shift indicated a combined effect of low pH and antimicrobial activity of EO. Furthermore, this blend of EO showed the ability to improve microbial protein synthesis in sheep (237). Higher microbial protein might be attributed to the enhanced post-ruminal protein supply and absorption. Moreover, it might possibly due to the reduction of protozoal counts as ruminal protozoa devour many bacteria and their protein flow toward the small intestine (237). Recently, Garcia et al. (238) revealed that the chemical composition of EO, especially proportion of oxygenated compounds, has a positive interaction with fermentation pattern and indicate promising potential regarding CH_4_ mitigation. However, EO have shown inconsistent effects on rumen microbes and feed degradability in different studies, owing to different types of EO used, their chemical composition, and their variable dietary and host responses (Tables 5, 6).

Supplementation of oregano EO at 4 and 7 g/d promoted the population of primary cellulolytic bacteria and ruminal fungi, respectively, in sheep (241). An *in vitro* study revealed that the inclusion of EO of *S. spicatum* in a high-concentrate diet significantly improved the rumen fermentation characteristics by reducing CH_4_ and NH_3_-N while promoting propionate concentration (252). Dietary supplementation of EO (coriander, geranyl acetate, and eugenol) reduced CH_4_ production up to 6% per cow/day and 20% less CH_4_ per kg of milk. It can be speculated that energy saved through this reduced CH_4_ production may be diverted toward milk production (253). A blend of EO (carvacrol, caryophyllene, p-cymene, cineole, terpinene, and thymol) altered the rumen functions by selectively promoting the growth of rumen bacteria (by decreasing *Firmicutes* while increasing *Bacteroidetes*) in calves (257). Essential oils of *Lippia turbinata* and *Tagetes minuta* have shown a 10-fold decrease of *in vitro* CH_4_ production coupled with modification of N metabolism in the rumen (249). Recently, a meta-analysis showed that dietary supplementation of a blend of EO (coriander, eugenol, and geraniol) increased the milk yield (3.6%), milk fat and protein (4.1%), and feed efficiency (4.4%), while decreasing DM intake (12.9%) and CH_4_ production (8.8%) during long term trial in dairy cattle (258).

Essential oil–cobalt complexes have shown positive effects in ruminants by enhancing productive performance while decreasing NH_3_ emissions (233). Likewise, synergetic effects of EO (thyme, mint, and savory) in a high-concentrate diet have been observed regarding desirable shifts in microbial fermentation and higher microbial protein yield in dairy cows (251). Increased feed efficiency and calcium homeostasis have been observed with supplementation of a plant bioactive EO blend (>80% menthol, eugenol, and anethol). Increased uptake of calcium and ammonium was also observed as a result of specific cation-transporting proteins expressed by the rumen. However, further investigations are needed to evaluate the fate of the absorbed nutrients, especially calcium and N (259). Recently, Zhou et al. (239) suggested that oregano EO (52 mg/L) in mature ruminants can modify ruminal fermentation and mitigate *in vitro* CH_4_ production through mediating ruminal bacteria (*Prevotella* and *Dialister*). Some studies involving supplementation of EO in ruminants have shown contrary findings as the feeding of oregano EO did not reduce CH_4_ yield together with no effect on animal performance and rumen fermentation (240, 248). These divergent findings may be partially explained by variable experimental conditions of studies including the type of diets, plant species, dose and type of EO, pH of rumen fluid, and host animal (260, 261).

Studies have suggested the use of a combination or blend of different EO as a better strategy to modulate rumen microbiome to manipulate rumen fermentation than using individual EO. This is mainly because each EO possesses a complex mixture of phytochemicals and their synergistic effects can lead to the synthesis of new compounds with a quite different bioactivity that could not be harvested with individual compounds (29, 262). Additionally, using a combination of phytochemicals is also advantageous for the host regarding the provision of various phytonutrients from different plant combinations. Moreover, the benefits of such a combination are its ultimate utility for using on large scale in the animal industry as a commercial feed additive to have an overall impact on the improvement of global animal production while mitigating GHG emissions.

## Future Implications

Rumen microbiome plays a critical functional role in N_2_ utilization, rumen feed fermentation, and CH_4_ production, ultimately influencing the production, health, and welfare in ruminants. Rumen microbes are highly active and can adapt to an extensive range of dietary fluctuations or host physiological conditions. Extensive literature supports the supplementation of phytogenic feed additives like saponin, tannins, and EO for the manipulation of rumen microbiome to modulate ruminal fermentation to increase VFA and decrease NH_3_ and CH_4_ production. Decreasing methanogenesis using dietary interventions at the expense of decreased VFA production is nutritionally adverse and unadvisable. Inhibition of enteric CH_4_ emission in ruminants is possible through the use of plant bioactive compounds; however, studies on the long-term effects of these compounds to reduce methanogenesis are essentially required. Studies summarized above clearly demonstrate that although phytochemicals possess a potent ability to modulate rumen microbiome and reduce methanogenesis *in vitro*, the observed *in vivo* effects varied greatly. Many factors, including variations of the chemical compositions of the compounds due to the differences in plant origin, growing conditions, and processing methods as well as different application methods, feeding conditions, and progressive adaptation of microbes for specific phytochemicals, contribute to this vast variability. Because of the complexity of these issues, it is difficult to conduct systematic and comprehensive evaluations of the efficacy and safety of these compounds for commercial applications in the animal industry. Therefore, controlling this variability is key to developing phytogenic substances as natural feed additives. This ideally should include all procedures from production, extraction, processing, and application. Optimization of different conditions during these steps can definitely help to address problems like inconsistency and transient and adverse effects of phytogenic feed additives in ruminants. Recent developments in molecular docking analysis and three-dimensional structure databases of phytochemicals have opened a new horizon for the discovery of putative functions (particularly antimicrobial and antimethanogenic) of different compounds by evaluating the structure affinity relationships with different microbes and their substrates. It will be of interest to identify potent phytochemicals based on their structural homology and binding affinity with functional proteins of rumen microbes particularly methanogenic archaea through molecular docking analysis first and then testing their biological activity *in vitro* and *in vivo*. This approach will not only help to find out new phytochemicals with potent activities but also help to understand their mechanism of action and exploit their synergistic effects and interactions with other compounds. Moreover, there is a dire need to exploit advances in molecular chemistry like encapsulation techniques to avoid ruminal degradation of phytochemicals and use their nanostructures to enhance their bioactivity and bioavailability, which seems to be an exciting area to explore their promising effects on the rumen microbiome.

It has been proposed that higher molecular weight compounds (such as polyphenols) were not dissolved well in water (263). Nanoparticles are formulated with hydrophobic groups inside and polar groups on the surface of particles and have shown to significantly enhance the solubility and bioavailability of less-water-soluble phenolic phytochemicals (264). Moreover, nanoparticles of plant extracts and EO have shown higher antioxidant and antimicrobial activities as compared with their crude extracts or EO (265). The higher antimicrobial activity of the nanoparticles or nanoemulsion of phytochemicals is related to the size of the nanoparticles or nanoemulsion droplets, which is in the subcellular size range. This allows the penetration of the nanoparticles or nanoemulsion droplets to the microbial cells leading to enhanced activity (265, 266). It is anticipated that the use of nanoparticles and nanoemulsions can potentially enhance the modulatory effects of phytochemicals on the rumen microbiome, subsequently leading to better health and performance of ruminants.

## Author Contributions

FH and CY: conceptualization. MAA, SS, and HME: data curation. CY: funding acquisition, project administration, supervision, and validation. FH, MSK, and MSR: investigation. MSR and CY: resources. FH and MSR: software. MAA, MSR, MSK, and FH: writing—original draft and formal analysis. MAA, FH, HME, and CY: writing—review and editing. All authors contributed to the article and approved the submitted version.

## Conflict of Interest

The authors declare that the research was conducted in the absence of any commercial or financial relationships that could be construed as a potential conflict of interest.
